# CD146 increases stemness and aggressiveness in glioblastoma and activates YAP signaling

**DOI:** 10.1007/s00018-022-04420-0

**Published:** 2022-07-05

**Authors:** Yuanke Liang, Daniëlle Voshart, Judith T. M. L. Paridaen, Nynke Oosterhof, Dong Liang, Arun Thiruvalluvan, Inge S. Zuhorn, Wilfred F. A. den Dunnen, Guojun Zhang, Haoyu Lin, Lara Barazzuol, Frank A. E. Kruyt

**Affiliations:** 1 Department of Medical Oncology, University of Groningen, University Medical Center Groningen, Hanzeplein 1, 9713 GZ Groningen, The Netherlands; 2 Department of Thyroid and Breast Surgery, Clinical Research Center, The First Affiliated Hospital of Shantou University Medical College, 57 Changping Road, Shantou, China; 3 Department of Radiation Oncology, University of Groningen, University Medical Center Groningen, Hanzeplein 1, 9713 GZ Groningen, The Netherlands; 4 Department of Biomedical Sciences of Cells and Systems, University of Groningen, University Medical Center Groningen, Hanzeplein 1, 9713 GZ Groningen, The Netherlands; 5 European Research Institute for the Biology of Ageing (ERIBA), University Medical Center Groningen (UMCG), University of Groningen, Groningen, The Netherlands; 6 Department of Biomedical Engineering, University of Groningen, University Medical Center Groningen, A. Deusinglaan 1, 9713 AV Groningen, the Netherlands; 7 Department of Pathology and Medical Biology, University of Groningen, University Medical Center Groningen, Hanzeplein 1, 9713 GZ Groningen, The Netherlands; 8 The Cancer Center and the Department of Breast Thyroid Surgery, Xiang’an Hospital of Xiamen University, 2000 East Xiang’an Rd, Xiamen, Fujian China

**Keywords:** MCAM, Glioblastoma stem cells, Cancer stem cells, EMT, Zebrafish, YAP

## Abstract

Glioblastoma (GBM), a highly malignant and lethal brain tumor, is characterized by diffuse invasion into the brain and chemo-radiotherapy resistance resulting in poor prognosis. In this study, we examined the involvement of the cell adhesion molecule CD146/MCAM in regulating GBM aggressiveness. Analyses of GBM transcript expression databases revealed correlations of elevated CD146 levels with higher glioma grades, IDH-wildtype and unmethylated MGMT phenotypes, poor response to chemo-radiotherapy and worse overall survival. In a panel of GBM stem cells (GSCs) variable expression levels of CD146 were detected, which strongly increased upon adherent growth. CD146 was linked with mesenchymal transition since expression increased in TGF-ß-treated U-87MG cells. Ectopic overexpression of CD146/GFP in GG16 cells enhanced the mesenchymal phenotype and resulted in increased cell invasion. Conversely, GSC23-CD146 knockouts had decreased mesenchymal marker expression and reduced cell invasion in transwell and GBM-cortical assembloid assays. Moreover, using GSC23 xenografted zebrafish, we found that CD146 depletion resulted in more compact delineated tumor formation and reduced tumor cell dissemination. Stem cell marker expression and neurosphere formation assays showed that CD146 increased the stem cell potential of GSCs. Furthermore, CD146 mediated radioresistance by stimulating cell survival signaling through suppression of p53 expression and activation of NF-κB. Interestingly, CD146 was also identified as an inducer of the oncogenic Yes-associated protein (YAP). In conclusion, CD146 carries out various pro-tumorigenic roles in GBM involving its cell surface receptor function, which include the stimulation of mesenchymal and invasive properties, stemness, and radiotherapy resistance, thus providing an interesting target for therapy.

**Supplementary Information:**

The online version contains supplementary material available at 10.1007/s00018-022-04420-0.

## Introduction

Glioblastoma multiforme, currently named glioblastoma (GBM), is the most common and lethal primary malignant brain tumor. Despite maximal initial resection followed by radiation and chemotherapy, the median survival with standard of care remains around 15 months [1]. Experimental evidence has indicated that GBM aggressiveness and recurrence are driven by a subset of cells displaying stem cell properties, such as unlimited self-renewal, high tumor-forming potential, and chemo- and radioresistance [2]. Great effort has been put into characterizing these GBM stem cells (GSCs), and unravelling the molecular pathways regulating stemness may thus yield new therapeutic targets for improving patient prognosis [3, 4].

Melanoma cell adhesion molecule (MCAM), also named cluster of differentiation 146 (CD146), is an integral membrane glycoprotein belonging to the immunoglobulin (Ig) superfamily that was originally discovered in metastatic melanoma and to be associated with poor prognosis [5, 6]. CD146 is a cell adhesion molecule that is likely activated via homophilic cell–cell or heterophilic cell–extracellular matrix (ECM) interactions [7]. For example, Laminin 411 was identified as a CD146 ligand that facilitates T cell entry into the central nervous system [8]. Moreover, CD146 was demonstrated to mediate tumor cell invasion, epithelial–mesenchymal transition (EMT), and metastasis in breast cancer [9]. Furthermore, we previously showed that CD146 confers tamoxifen and cisplatin resistance in breast cancer cells and that its expression is associated with poor prognosis in breast cancer patients [10, 11].

The YAP/TAZ transcriptional activators, known to be regulated by the Hippo signaling pathway, have been identified as primary sensors of a variety of signals generated by cell–cell contacts, cell–ECM adhesion, mechanical stress, as well as alterations in the metabolic state of the cell [12, 13]. Deregulation of the Hippo/YAP pathway significantly contributes to tumorigenesis, metastasis, and therapeutic resistance and is associated with poor prognosis in glioma, colon, breast, and ovarian cancer patients [10, 13, 14]. The core components of the Hippo pathway, Mammalian Ste20-like kinases 1/2 (MST1/2), and large tumor suppressor 1/2 (LATS1/2) kinases are well established; however, knowledge of the various upstream regulatory mechanisms is still expanding.

In human glioma patient samples, increased CD146 expression has been correlated with higher tumor grades and was suggested to be a potential diagnostic and therapeutic target [15]. However, the role of CD146 in GBM has not been further substantiated and the underlying molecular mechanisms are incompletely understood. Here we examined the potential of CD146 to serve as a prognostic factor in GBM and examined its importance for regulating GBM aggressiveness using various in vitro and in vivo models. We show that CD146 enhances multiple aggressive features of GBM, such as stemness, mesenchymal and invasive properties, and radioresistance. Interestingly, we also identified YAP as a novel downstream target of CD146.

## Materials and methods

### Cell culture

Human U-87MG (RRID:CVCL_0022) cells were obtained from American Type Culture Collection and U-251MG (RRID:CVCL_0021) cells from the CLS Cell lines Service GmbH (Eppelheim, Germany). U-87MG and U-251MG were cultured in 10% FCS and 1% l-glutamine DMEM medium. GSC23, kindly provided by dr. Krishna Bhat (Department of Pathology, MD Anderson Cancer Center, Texas University, USA), GG6, GG9, GG12, and GG16 were generated from GBM surgical samples and have been described before [16, 17]. These cells were cultured as neurospheres in Neurobasal-A medium with 2% B27 supplement, 20 ng/ml bFGF, 20 ng/ml EGF, and 1% l-glutamine or as adherent cells on 2% matrigel-coated dishes. GBM neurospheres were differentiated with 10% FCS culture medium. Cell cultures were maintained for around 30 passages for regular cell lines and for 20 passages for neurospheres. Cell lines are regularly tested for mycoplasma by PCR and authenticated by STR profiling (Baseclear, Leiden, The Netherlands).

### TCGA and CGGA dataset analyses

CD146 expression and clinical data, such as tumor grade, histological type, IDH1 mutation status, MGMT methylation status, survival, and outcome, were obtained and analyzed from the TCGA (The Cancer Genome Atlas, HG-UG133A microarray and GBMLGG RNA-seq data). CD146 expression and GBM patients’ clinical data, such as treatment (radiotherapy, chemotherapy), survival, and outcome, were downloaded from CGGA data portal (Chinese Glioma Genome Atlas, http://www.cgga.org.cn/). R language (edgeR package, R version 3.51) and GraphPad Prism 8.0.2 software were used to analyze and plot the results.

### Western blotting

Proteins were extracted from cells with RIPA buffer (Thermo Fisher Scientific, Breda, The Netherlands) containing 1% protease inhibitor and 1% phosphatase inhibitor (Thermo Fisher Scientific). The Pierce BCA protein assay kit (Thermo Fisher Scientific) was used to quantify total protein. Protein samples were separated on SDS-PAGE gels, transferred to PVDF membranes, and after blocking in 5% nonfat milk in TBST buffer subjected to incubation with different primary antibodies. After overnight incubation with primary antibodies (see Supplementary Table S1) at 4℃, the blots were incubated with HRP-conjugated secondary antibody for one hour and visualized using ECL Substrates (Roche, Roche Diagnostics Nederland B. V., Flevoland, The Netherlands).

### Neurosphere formation assay

Neurospheres were washed with PBS followed by accutase (Sigma-Aldrich, Zwijndrecht, The Netherlands) treatment to dissociate cells. The single cell suspension was sorted based on forward and side scatter pattern using SH800S Cell Sorter (Sony Biotechnology, Weybridge, UK). 10, 20, 40, or 80 cells/well were seeded in 96-well plates in 150 μl medium. The number of neurospheres per well was counted after 2 to 3 weeks. Each condition was performed in triplicate.

### Generation of CD146 overexpression and CRISPR/Cas9 knockout cell models

The pCMV-CD146/ GFP plasmids and corresponding empty vector pCMV-GFP were purchased from Sino Biological Inc. (Beijing, China). Control and exon1 and exon2 directed gRNAs for CD146 were cloned into pSpCas9(BB)-2A-GFP(PX458) (Addgene Teddington, UK), according to Ann Ran et al. [18]. DNA oligonucleotides for CD146 guide were exon-3-1_GCTCAGCCTCCAGGACAGAG and guide-exon-3-2_GGAGAGGCCGCACTTCAGAA. Cells were transfected with the plasmids using Lipofectamine 3000 reagent (Thermo Fisher Scientific) according to the manufacturer's instructions. After 48 h, transfected cells were dissociated and GFP positivity cells were sorted by using SH800S Cell Sorter. GG16 transfected cells were maintained in medium with 75 μg/ml Hygromycin and single cell sorted GSC23 control and CD146-ko cells were plated in 96-well plates for expansion. CD146 overexpressing or ablated cells were determined by Western blotting.

### Immunofluorescence microscopy

Cells were seeded and grown to 60% confluence in Millicell EZ 8-well glass slides (Merck Millipore, Germany) and fixed with 4% paraformaldehyde. Subsequently, cells were treated with 0.5% Triton X-100, blocked with 10% BSA for 20 min, incubated with primary antibodies (see Supplementary Table S1) overnight at 4℃, and incubated with secondary antibodies (Alexa Fluor 488 goat anti-mouse IgG1 and Alexa Fluor 568 goat anti-rabbit IgG; Invitrogen, USA) at room temperature for 1 h. Slides were mounted in Vectashield with DAPI (Life Technology, NY, USA). Images were visualized and captured with EVOS XL Core Cell Imaging System (Thermo Fisher Scientific).

### Migration and invasion assays

Transwell assays were conducted to examine the cell migration and invasive capacity, as described previously [30]. Briefly, cells were seeded in upper transwell chambers (8 μm pore size; Corning, USA) with 0.1% FBS medium. Medium with 10% FBS was added to the lower chamber. After culturing for 24 h, cells were fixed with 4% paraformaldehyde and stained with 0.1% crystal violet. The number of cells from 5 fields in each well was counted. Each experiment was performed in triplicate.

### RNA isolation and qRT-PCR

Cells were treated as indicated and RNA was isolated from cell pellets using RNeasy Mini Kit (Qiagen, Germany) following the manufacturer’s protocol. Reverse transcription was performed using the iScript™ cDNA Synthesis Kit (BioRad, Veenendaal, The Netherlands) according to the manufacturer’s instructions. qRT-PCR was performed in triplicate using the iTaq Universal SYBR Green Supermix (BioRad) in CFX96 TouchTM Real-Time PCR Detection System C1000 Thermocycler (BioRad). Primer sequences for qRT-PCR are listed in Supplemental Table 2. PCR reactions were performed at 50 °C for 2 min and 95 °C for 2 min, followed by 40 cycles of 95 °C for 15 s and 60 °C for 1 min. Cycle threshold (*C*_T_) values for individual reactions were obtained using CFX Manager Software (BioRad). To determine relative gene expression levels, the *C*_T_ values were normalized to the house-keeping gene GAPDH using the Δ*C*_T_ method.

### Clonogenic assay

GBM cells as indicated were seeded in 6-well plates for 24 h followed by mock or 2, 4, or 6 Gy ionizing radiation (IR). After 2 weeks, colonies were fixed and stained with crystal violet. Colonies consisting of at least 50 cells were counted using Start VSpot-Spectrum (Autoimmun Diagnostika GMBH, Germany).

### Generation of human cortical organoids and fusion into GBM-cortical assembloids

The induced pluripotent stem (iPS) cell line EH1 [19] was used to generate cortical organoids according to the protocol of the Pasca laboratory [20, 21]. Generated organoids were fused with GSC23 control or GSC23-CD146-ko neurospheres to form GBM-cortical assembloids. Refer to supplementary materials for details.

### Zebrafish xenograft experiments

Zebrafish were housed and handled according to European animal welfare regulations and standard protocols. Zebrafish embryos used were progeny of transparent Casper fish. CellTracker™ Green CMFDA Dye (Invitrogen, The Netherlands)-labeled GSC23-WT, or GSC23-CD146-ko single cell suspensions were injected in 1-day post-fertilization zebrafish embryos into the perivitelline space (PVS) between the periderm and yolk sac, as described before [22]. Tumor cell dissemination was determined after 2, 24, and 72 h and images of live embryos were taken with a fluorescence stereomicroscope. For further imaging analyses zebrafish xenografts were fixed in 4% paraformaldehyde, stained for human nucleoli, counterstained with DAPI, mounted on glass bottom dishes, and imaged on a confocal microscope. For quantification and categorization of tumor morphology, the green channel nucleoli images were converted to inverted gray LUT, and scored blindly by two investigators. The data presented are results from 3 independent experiments. Refer to supplementary materials for details.

### Statistical analysis

Each experiment was repeated at least 3 independent times unless otherwise indicated. Data are presented as the mean ± SEM, and Student’s *t* test with *P* < 0.05 was considered statistically significant.

## Results

### CD146 expression in glioma patients and in GBM cells

**Fig. 1:**
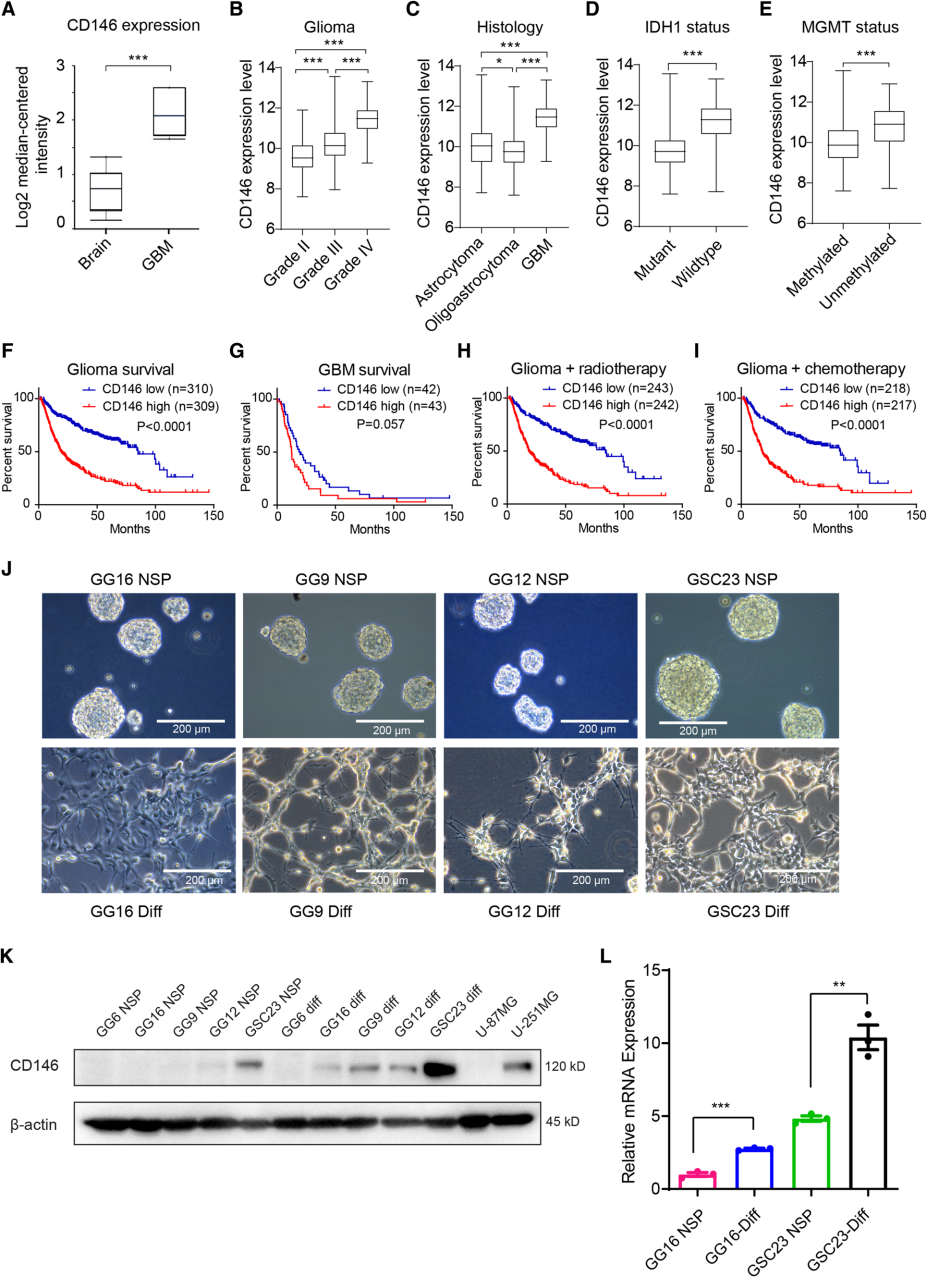
CD146 expression in glioma patients and in GBM cells. **A** Comparison of CD146 mRNA expression in normal human brain to GBM tissues using TCGA brain cancer database. Box plots were derived from gene expression data compared in *ONCOMINE* (*p* = 1.30E^−4^, fold change: 2.675). **B** Relative CD146 mRNA levels in grade II, grade III, and grade IV glioma; in astrocytoma, oligoastrocytoma, and GBM (**C**); in IDH-mutant glioma vs IDH wildtype (**D**); MGMT promoter methylated glioma vs the unmethylated subtype (**E**). **F** Kaplan–Meier plots derived from CGGA database showing significant differences in overall survival between low and high CD146 expression in glioma patients (**G**); Kaplan–Meier plot showing overall survival between low and high CD146 expression of GBM patients (**H, I**). Kaplan–Meier plot showing overall survival between low and high CD146 expression of glioma patients who received radiotherapy (± chemo) (**H**) and chemotherapy (± RT) (**I**). **J** Representative phase contrast microscopic images of indicated GBM neurospheres (NSP) cultured in serum-free medium (upper) or adherent serum-differentiated (Diff) counterparts (lower) (scale bars = 200 μm). **K** Western blots showing variable CD146 protein expression in GBM neurospheres that is increased upon serum differentiation. GBM cell lines U-87MG and U-251MG are also included. **L** Relative CD146 mRNA levels determined in by qRT-PCR in GBM neurospheres and serum-differentiated cells. Data represent the mean of triplicate experiments ± SEM, *p < 0.05; **p < 0.01; ***p < 0.001 by Student’s *t* test

To determine the expression of CD146 in GBM, we conducted an analysis of the GBM TCGA database. CD146 transcript levels were significantly elevated in GBM compared with normal brain tissue (Fig. 1A). CD146 transcript levels also positively correlated with glioma grade and subtype with GBM having the highest levels (Fig. 1B, C). IDH1-wildtype (IDH1-wt) and unmethylated MGMT had higher CD146 levels compared with IDH-mutant (IDH-mut) and methylated MGMT (Fig. 1D, E), indicating that elevated CD146 expression is associated with aggressive GBM properties. High CD146 expression also correlated with worse overall survival (OS) in glioma and GBM (Fig. 1F, G), and was associated with worse response to radio- and chemotherapy (Fig. 1H, I, Supplementary Fig. 1A, B). CD146 mRNA levels showed no clear association with a particular GBM subtype, classical, mesenchymal, neural, and proneural, and levels were considerable variable within each subtype (not shown).

Next, CD146 expression was examined in the available GBM patient-derived neurosphere models, GG16, GG9, GG12, and GSC23, which can be serum-differentiated leading to adherent growth (Fig. 1J). Western blotting showed variable levels of CD146 with detectable levels in GSC23, and very low or absent protein in GG12, GG9, and GG16 neurospheres. Additionally, U-87MG cells had no detectable CD146 expression, whereas U-251MG cells showed higher expression. Interestingly, CD146 protein levels were upregulated upon serum differentiation, GSC23 showing again the highest levels compared to the other cells (Fig. 1K). In addition, previously generated mRNA-seq data from GG16 and GSC23 cells [23] were consistent with the protein data showing higher levels of CD146 transcripts in GSC23 cells and levels increasing upon serum differentiation (Fig. 1L).

Since differentiated neurospheres showed upregulated CD146 expression, we explored if either cell adherence, differentiation or both were mediating this effect. For this, GSC23 cells were cultured for different time periods under stem cell maintaining conditions, either as neurospheres or adherently cultured in matrigel-coated plates, and in the presence or absence of serum (Supplementary Fig. 1C). Neurosphere cultured cells showed relatively low CD146 protein levels that strongly increased within 24 h upon plating in matrigel-coated wells and by serum differentiation (Supplementary Fig. 1D). Thus, cell adhesion of GSCs mediated by ECM or differentiation increases CD146 expression.

Together these results show that CD146 expression levels correlate with glioma grade and subtype, worse OS, and poor outcome after radio- and chemotherapy. The in vitro models reveal variable expression of CD146 that was enhanced upon both ECM–cell adhesion and differentiation.

### CD146 promotes a mesenchymal phenotype and stimulates GBM cell invasion

**Fig. 2:**
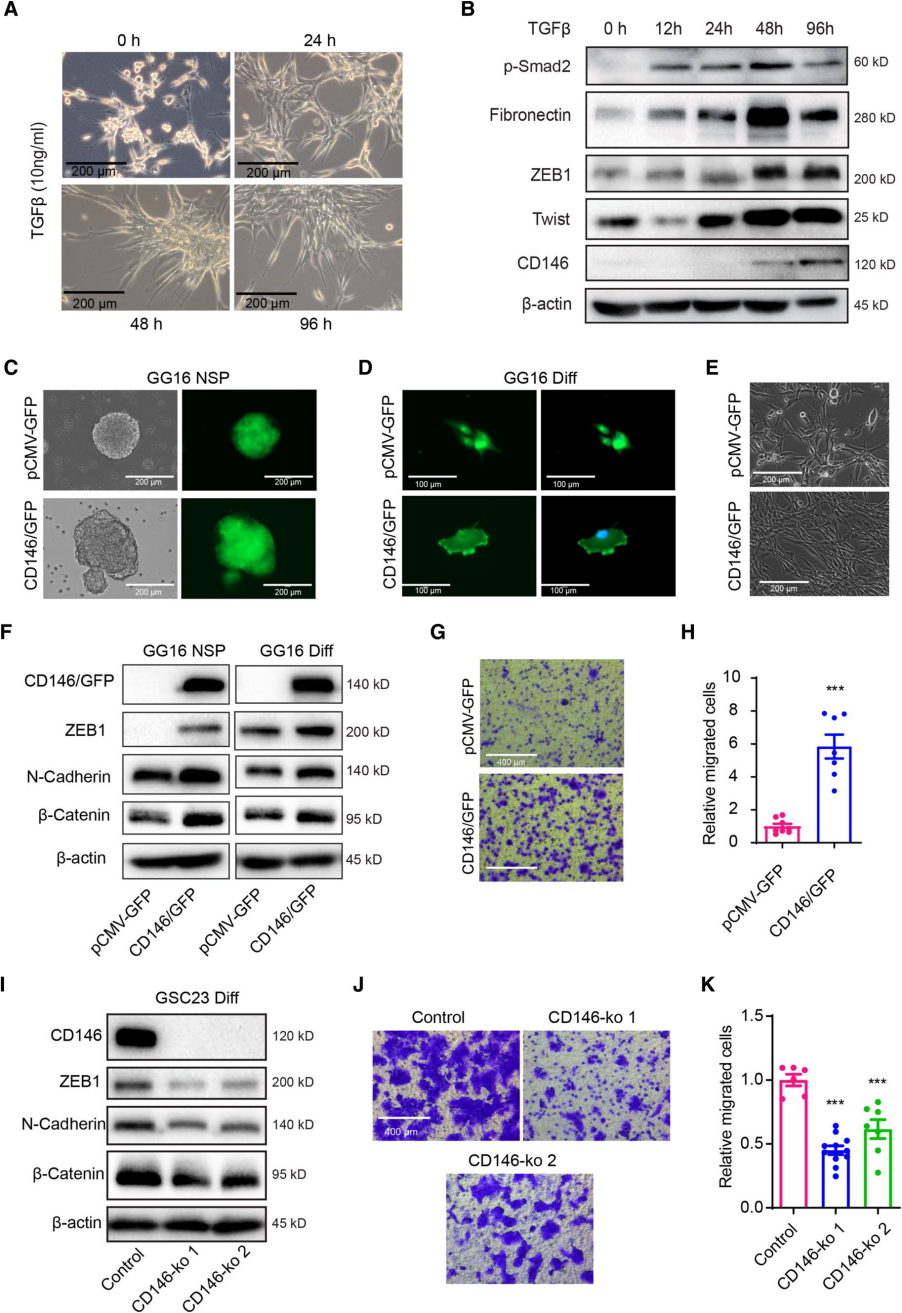
CD146 regulating mesenchymal properties in GBM cells. **A** Representative phase contrast microcopy images of U-87MG cells treated with TGFβ (10 ng/ml) for 0 h, 24 h, 48 h, and 96 h showing induction of a spindle-shaped, stretched out mesenchymal morphology (scale bars = 200 μm). **B** Western blots showing TGFβ-induced expression of p-Smad2 and mesenchymal markers Fibronectin, ZEB1, Twist1, and CD146 in U-87MG cells. **C, D** Phase contrast and immunofluorescent microscopic images of GG16 cells ectopically overexpressing GFP (control) or a CD146/GFP fusion protein in spheroids and serum-differentiated cells (scale bars = 200 μm). **E** Phase contrast microscopic images of differentiated GG16 or GG16 CD146 overexpressing cells (scale bars = 200 μm). **F** Overexpression of CD146/GFP in GG16 cells is accompanied by increases in ZEB1, N-cadherin, and β-catenin protein levels as measured by Western blotting. **G** Transwell migration assays demonstrate increased migration in CD146/GFP overexpressing GG16 cells compared to GG16-GFP control cells (scale bars = 400 μm). **H** Representative images of cells on Transwell membranes and quantified data are presented. Data represent the mean of triplicate experiments ± SEM, ***p < 0.001 by Student’s *t* test. **I.** Two independently generated GSC23-CD146-ko cells cultured in serum differentiation conditions have reduced expression of mesenchymal markers ZEB1, N-cadherin, and β-catenin compared to control cells as detected by Western blotting. **J** Representative images of Transwell migration assay membranes (scale bars = 400 μm) and **K** quantified data comparing GSC23 control and GSC23-CD146-ko cells, ***p < 0.001 by Student’s *t* test

CD146 has been implicated in EMT in several cancers [9]. Previously, we showed that TGF-ß induces mesenchymal transition (MT) in U-87MG cells [16]. TGF-ß exposure resulted in a time-dependent change in cellular morphology acquiring a spindle-shaped, elongated and stretched appearance (Fig. 2A), as reported earlier [16]. Western blotting showed TGF-ß-induced pSmad2 activation and time-dependent increased expression of the EMT transcription factors ZEB1 and Twist, together with increased expression of Fibronectin and importantly of CD146 (Fig. 2B).

Next, we examined the effect of CD146 on the malignant phenotype of patient-derived GBM neurospheres. For this pCMV-CD146/GFP and control pCMV-GFP were stably transfected in GG16 neurospheres that have low endogenous CD146 expression (see also Fig. 1K). As expected, we found overexpression of the fusion protein CD146/GFP that was mostly located at the cell membrane, while GFP staining in control GG16 cells was uniformly distributed in the cytoplasm and nucleus of both neurospheres and differentiated cells (Fig. 2C, D). Serum-differentiated GG16-CD146/GFP cells had a more spindle-shaped and elongated mesenchymal morphology than GG16-GFP control cells (Fig. 2E). Ectopic overexpression of CD146 significantly increased ZEB1, β-Catenin, and the mesenchymal marker N-Cadherin in GG16 cells (Fig. 2F). Furthermore, GG16-CD146/GFP cells showed a sixfold increase in migration potential compared to control cells in transwell assays, which is in agreement with an enhanced mesenchymal state (Fig. 2G, H). Thus, CD146 is involved in MT and can enhance migration in the GG16-CD146/GFP overexpression model.

In order to further study CD146 functioning we generated CD146 knockouts (ko) in GSC23 cells that displayed relatively high endogenous CD146 levels (see also Fig. 1K). Western blotting confirmed effective CD146-ko in two selected clones that were associated with reduced levels of ZEB1, ß-Catenin, and N-Cadherin in serum-differentiated GSC23 cells (Fig. 2I). Moreover, CD146 ablation reduced migration ability in GSC23-CD146-ko cells compared to controls (Fig. 2J–K). Together, these results indicate that CD146 stimulates the mesenchymal and migratory properties of GBM cells.

### CD146 enhances migration and invasion of GSC23 cells in a GBM-cortical assembloid model

**Fig. 3:**
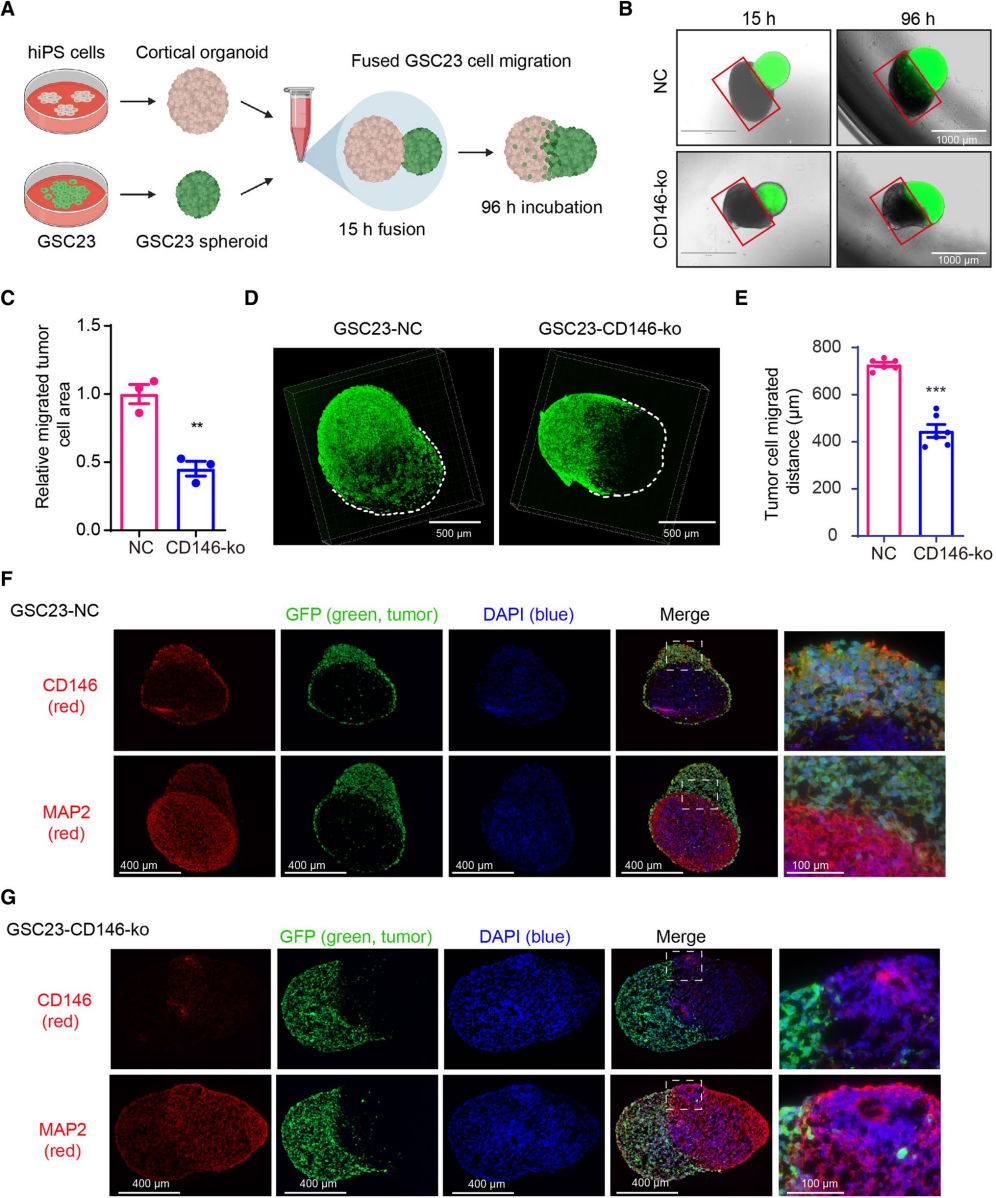
GSC23-CD146 knockout cells have reduced migration/invasion capacity in a GBM-cortical assembloid model. **A** Schematic outline of the generation of GBM-cortical assembloids to determine migration and invasion capacity. **B** Representative fluorescent microscopic pictures of GSC23-NC (control) and GSC23-CD146-ko (green) GBM-cortical assembloids at different time points after fusion (scale bars = 1000 μm). The red squares indicate the organoid area largely negative for GFP positive cells, and were used to quantify migration/invasion of GSC23 cells (see methods). **C** Quantification of migration/invasion by determining ratios GFP positive vs. negative areas, indicating reduced migration/ invasion of GSC23 CD146-ko cells. **D** Representative confocal immunofluorescent microscopic 3D images of the neurosphere-cortical assembloids 4 days post-fusion. **E** Distance quantification of migrated GFP positive tumor cell in the GBM-cortical assembloid. Immunofluorescence microscopic images of GFP-labeled (green: GSC23 cells) GSC23 (**F**) and GSC23-CD146-ko GBM-cortical assembloids (**G**) stained for CD146 and the neuronal marker MAP2 (labeled red). Nuclei (blue) were stained with DAPI. Scale bars = 400 μm (left), scale bars = 100 μm (right). **p < 0.01, ***p < 0.001 by Student’s *t* test

The effect of CD146 on GSC23 aggressiveness was further examined by making use of a 3D human induced pluripotent stem cell (iPS)-derived cortical organoid system resembling the cortex. Similar cerebral organoids have been used as a brain-mimicking system to study the growth of GSCs [24]. GFP-labeled GSC23 control and GSC23-CD146-ko neurospheres were fused with cortical organoids to form the here called GBM-cortical assembloids, and GSC cell invasion was followed over time (Fig. 3A). By using confocal immunofluorescent microscopic 3D imaging, we found that neurospheres rapidly fused with the organoid and after 4 days control GSC23 almost completely engulfed the cortical organoid, whereas CD146-ko counterparts showed reduced migration/ invasion (Fig. 3B). Quantification of this process by determining the ratio tumor cell area to the tumor-free area showed an approximately twofold reduced infiltration of GSC23-CD146-ko cells after 96 h (Fig. 3C). Confocal immunofluorescent microscopy was used to image the 3D structure of the GBM-cortical assembloids, and we found that the migrated distance of GFP-labeled GBM cells was significantly decreased in GSC23-CD146-ko cells (Fig. 3D, E). GBM-cortical assembloid slices were examined for expression of CD146 and the neuronal marker MAP2. MAP2 was specifically found in the cortical organoid, whereas, as expected, CD146 expression was detected in GSC23 control cells and mostly absent in GSC23-CD146-ko cells (Fig. 3F, G). GFP expression showed that GSC23 cells engulfed the majority of the cortical organoid volume, while less engulfment was visible in the CD146-deficient cells.

Together, these results further illustrate the stimulatory effect of CD146 on GBM cell infiltration in a human cortex mimicking model.

### CD146 enhances migration/ invasion of GSC23 cells in a zebrafish xenograft model

**Fig. 4:**
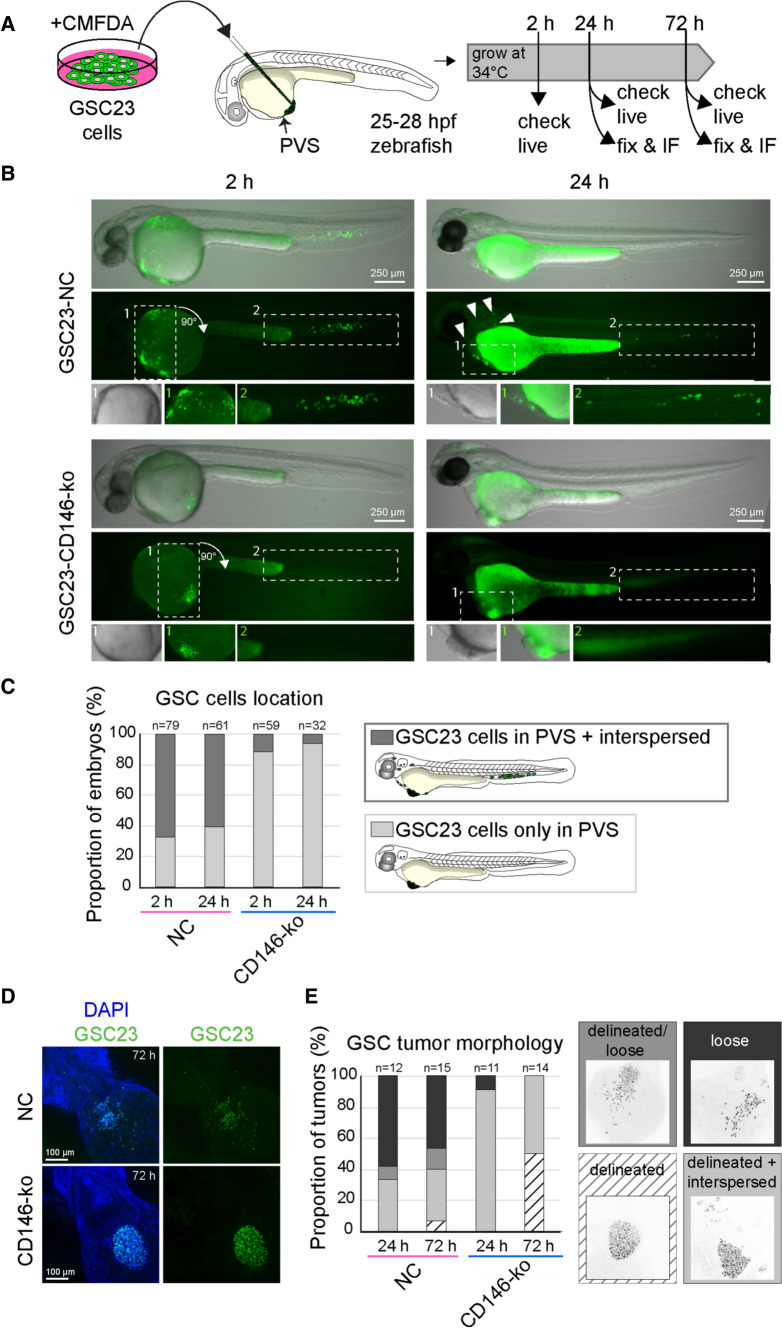
GSC23-CD146 knockout cells showing reduced migration/invasion in a heterotopic xenograft zebrafish model. **A** Schematic outline of the zebrafish PVS xenograft procedure. GSC23 control and GSC23-CD146-ko neurospheres were dissociated and stained with a green fluorescent dye (CMFDA) prior to injection into the perivitelline space (PVS) at 25–28 h post-fertilization (hpf) in *Casper* (transparent) zebrafish embryos. Xenografted embryos were assessed and fixed for further analysis at several time points after transplantation. **B** Representative fluorescence stereomicroscope images of a live zebrafish embryo containing either xenografted GSC23 control (top row) or CD146-ko (bottom row) cells at 2 hpt (left column) and 24 hpt (right column). Shown are overlays of transmission light/fluorescence (top image), fluorescence channel only (middle image), and magnifications of indicated regions of the embryo (bottom images). Arrowheads indicate sparse individual GSC23 cells. **C** Qualitative assessment of the proportion of embryos with differential location of control (pink) versus CD146-ko (blue) GSC23 cells (see schematic drawing). Light gray indicates embryos with cells only in the PVS, dark gray indicates embryos harboring cells in the PVS and in the embryo proper (tail, trunk, head). Results are from *N* = 3 independent experiments with total n of embryos indicated above the bars, in which embryos that died between 2 and 24 h or without successful engraftment at 24 h were excluded from the 24 h bars. **D** Representative maximum projection confocal images of fixed xenografted embryos at 3 dpt with nuclei stained with DAPI (blue) and anti-human nucleoli antibody (green) to visualize the GSC23 cells. The images show a ventral view of the jaw, heart, and anterior yolk region of the embryos where the PVS tumors are located. Anterior is to the top left and posterior to the bottom right. **E** Qualitative assessment (categorization) of GSC23 PVS tumor morphologies in control (pink) and CD146-ko (blue) xenografts at 1 and 3 dpt. These categories were as follows: (1) delineated (white, striped), defined as compactly packed tumor with defined border with fewer than 10 cells outside the tumor; (2) delineated + interspersed (light gray), a compact delineated tumor with more than 10 interspersed or clustered cells outside the tumor; (3) delineated/loose (middle gray), a tumor that is partially compact with clear boundary and partially loose; and (4) loose (dark gray), a tumor with clear intercellular distances and absence of defined tumor boundary (**E**). Results are from *N* = 3 independent experiments with total n of embryos indicated above the bars. Scale bars in **B** = 250 μm; scale bars in *D* = 100 μm

To corroborate the role of CD146 in migration and invasion in an in vivo setting, a heterotopic xenograft model was established in zebrafish embryos. In this model, around 250 GSC23 or GSC23-CD146-ko cells were transplanted into the PVS of 1-day post-fertilization zebrafish embryo (Fig. 4A). This model allowed us to assess tumor cell migration and invasion, as previously demonstrated by others [25–27]. The xenografted embryos were monitored by microscopy at several time points for tumor morphology and dissemination (Fig. 4A). Within 4 h of transplantation control GSC23 cells had reached the embryo circulation, as a subset of labeled cells was found in the tail (caudal hematopoietic tissue) and in some cases also sparsely in the trunk and head (Fig. 4B, left column, top panel, Fig. 4C). In contrast, CD146-ko cells remained at the initial site of injection and rarely showed cases where cells had entered the circulation (Fig. 4B, left column, bottom panel, Fig. 4C). At 24 h post-transplantation (hpt) a similar pattern was observed, with only a subset of GSC23 cells remained in the PVS and more cells located outside of the PVS region in the majority of embryos (mainly in the tail; Fig. 4B, right column, top panel, Fig. 4C). In general, the total number of cells visible within the embryo was lower at 24 hpt compared to immediately after transplantation (Fig. 4B, top left and right panels), which suggests that control GSC23 cells entering the circulation might be cleared through cell death or macrocytosis. In contrast, GSC23-CD146-ko cells were clearly visible in the PVS (see magnifications in Fig. 4B, right column, bottom panel) and rarely cells outside the PVS were detected (Fig. 4C). These results indicate that CD146 depletion caused a reduction of GSC23 cell migration and invasion in the zebrafish xenograft model.

Next, the morphology of the xenografted tumors was examined in more detail by human-specific nucleoli staining and confocal imaging of fixed xenografted embryos at 24 and 72 hpt (Fig. 4D, E). At 72 hpt, images showed that the GSC23 cell masses within the PVS were dispersed or loose, whereas CD146-ko cell masses often formed a clearly delineated tumor (Fig. 4D). To quantify this, the confocal images were categorized into four different PVS tumor morphologies (Fig. 4E). Around 60–70% of the embryos implanted with control GSC23 cells showed a partially or fully loose morphology at 24 and 72 hpt. In contrast, at 24 hpt, 90% of CD146-ko xenografts showed clearly delineated tumors with few scattered cells or small cell clusters outside of the main tumor (Fig. 4E). At 72 hpt, clearly delineated tumors with just a few scattered cells outside of the main tumor made up 50% of the CD146-ko xenografts. Although we have not tracked individual GSC23 cell movements in individual embryos, we infer that differences in cell–cell adhesion and migration/invasion properties contribute to the observed reduced dissemination of CD146-ko cells in vivo.

### CD146 promotes stemness in GBM

**Fig. 5:**
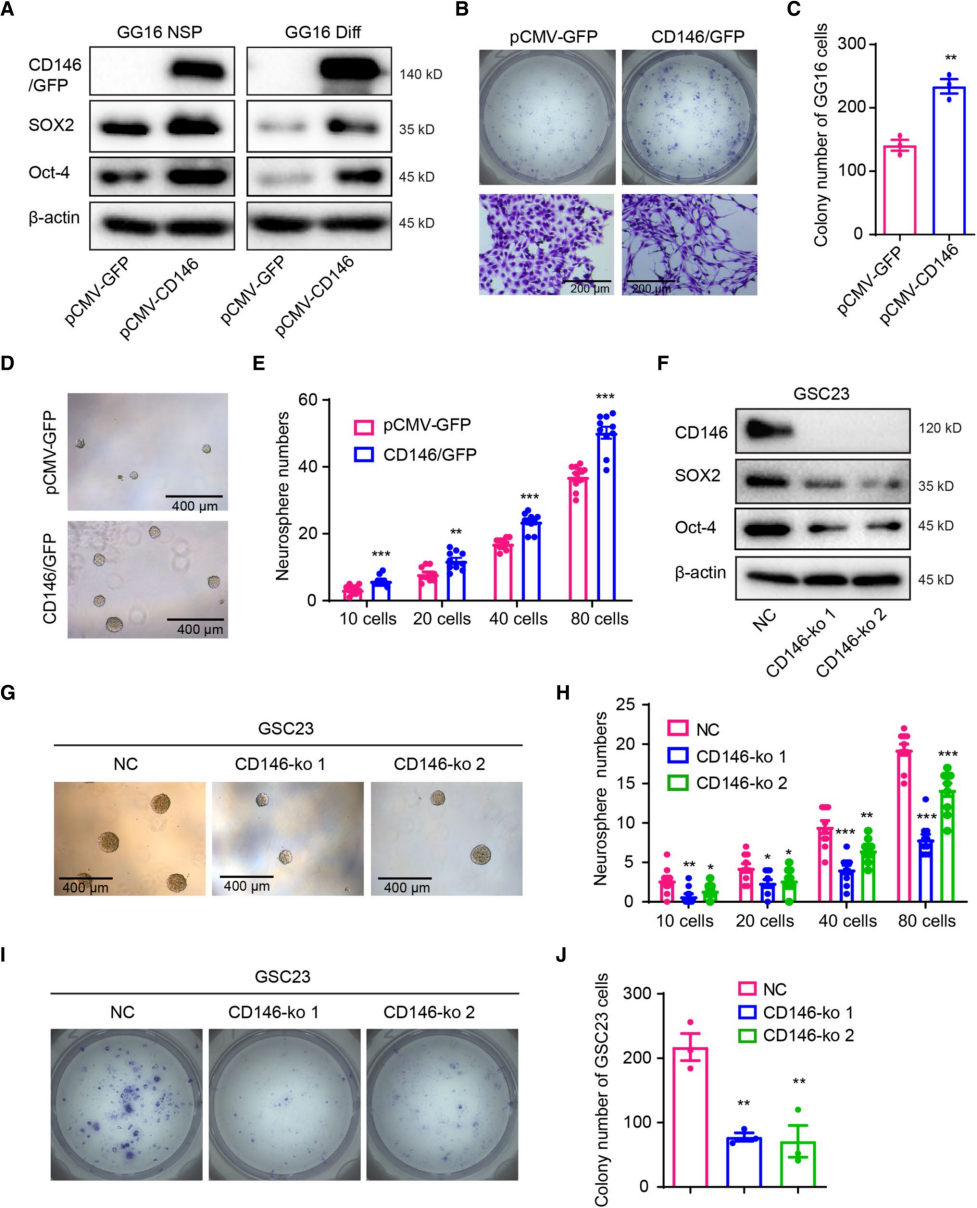
CD146 promotes stemness in GBM cells. **A** Western blots showing increased expression of SOX2 and Oct-4 in CD146/GFP overexpressing GG16 neurospheres (NSP) and differentiated cells (Diff). **B** Representative images of GG16 control and GG16-CD146/GFP colonies in colony formation assay showing more dispersed cells in CD146 overexpressing cells (scale bars = 200 μm). **C** Quantified data of colony numbers in GG16 control compared with CD146/GFP overexpressing GG16 cells for 2000 cells plated. **D, E** Representative images (scale bars = 400 μm) and quantified data of GG16 control or CD146/GFP overexpression neurospheres in limiting dilution assays. **F** Western blots showing reduced SOX2 and Oct-4 expression in two different GSC23-CD146-ko cells compared to control serum-differentiated cells. **G, H** Representative images and quantified data of GSC23 control and two different GSC23-CD146-ko neurospheres in limiting dilution assay. CD146 loss reduces neurosphere formation potential (scale bars = 200 μm). **I, J** Representative images and quantified data of differentiated GSC23 control vs GSC23-CD146-ko in colony formation assays. CD146 loss reduces colony formation potential in differentiated GSC23 cells. *p < 0.05; **p < 0.01; ***p < 0.001 by Student’s *t* test

To explore a possible effect of CD146 on stemness regulation, we determined the expression of the stem cell transcription factors SOX2 and Oct-4. SOX2 and Oct-4 protein levels were upregulated in GG16-CD146/GFP neurospheres compared to empty vector control cells and expression remained high after serum-induced differentiation, suggesting impaired differentiation (Fig. 5A). Indeed, serum-differentiated GG16-CD146/GFP cells had increased colony forming potential when compared to control cells (Fig. 5B, C). As observed before, GG16-CD146/GFP colonies consisted of cells with a more mesenchymal morphology. In addition, neurosphere formation assays demonstrated that CD146 overexpression significantly enhanced neurosphere formation potential in GG16 cells (Fig. 5D, E).

The involvement of CD146 in regulating self-renewal and proliferation potential was also examined in GSC23-CD146-ko cells and a strong decrease of SOX2 and Oct-4 expression was found compared to control cells (Fig. 5F). In addition, in the GBM-cortical assembloid model, CD146-deficient GSC23 cells showed less homogeneous and reduced SOX2 levels compared to CD146-proficient control (Supplementary Fig. 2A–C). Besides, SOX2 was also detected in ventricular zone-like structures in the cortical organoids known to contain neuronal progenitor cells [20, 21]. Neurosphere formation and colony formation assays demonstrated reduced neurosphere formation potential and proliferation capacity in GSC23-CD146-ko cells (Fig. 5G–J). Together, these findings indicate that CD146 stimulates stemness and proliferation potential in GBM cells.

### CD146 enhances GBM radioresistance

**Fig. 6:**
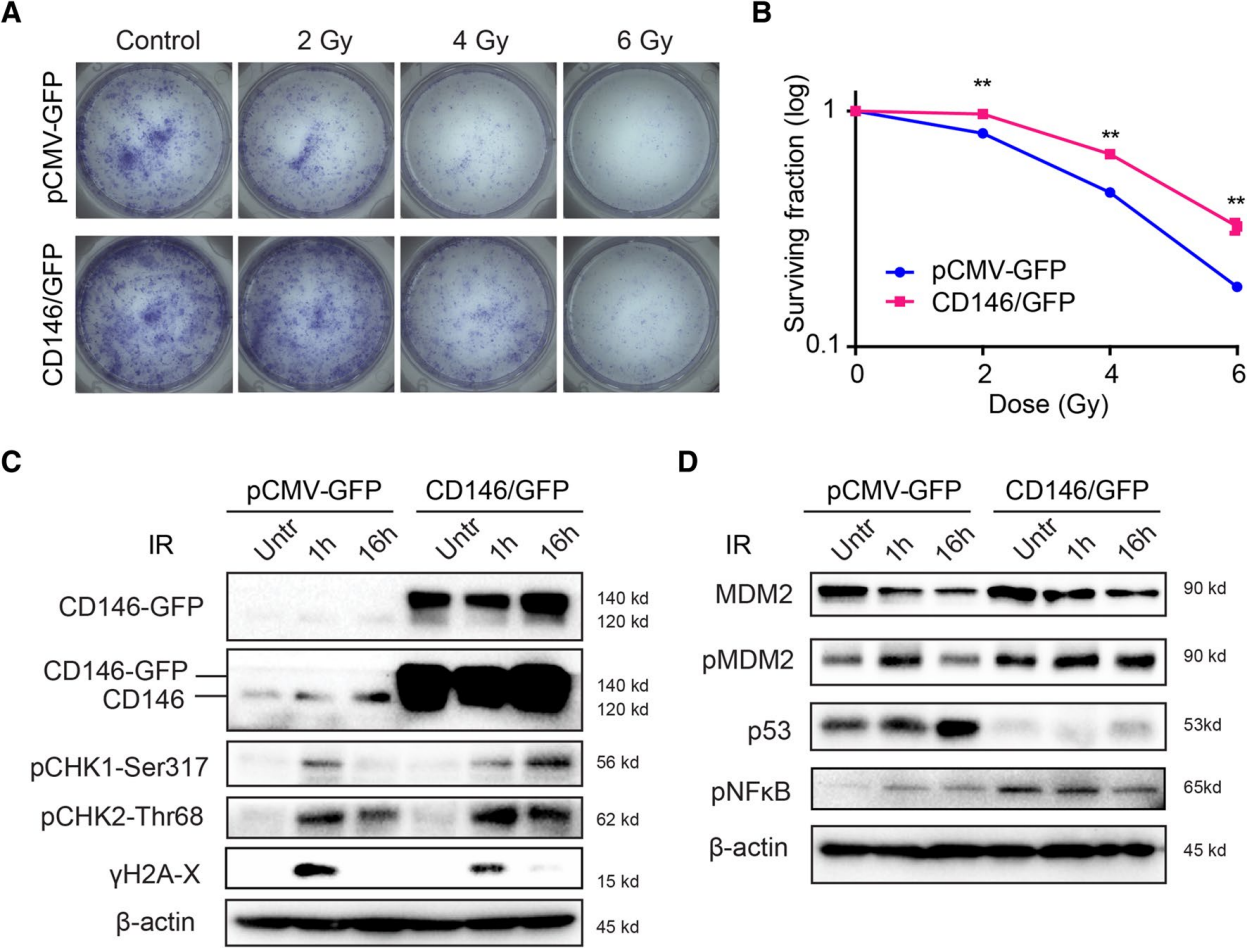
CD146 increasing radioresistance in GG16 cells. **A, B** GG16 control (pCMV-GFP) and CD146/GFP overexpressing cells were untreated or exposed to different doses of ionizing radiation (IR) and colony forming potential was monitored. Representative images of plates with colonies and survival curves are shown, **p < 0.01 by Student’s *t* test. **C** Western blots showing effect of 4 Gy IR in time on expression of CD146 and indicated DDR, and cell cycle regulatory proteins in GG16 GFP control and GG16-CD146/GFP cells cultured as differentiated cells. Endogenous CD146 expression is induced by radiation, and overexpression of CD146/GFP is associated with prolonged CHK1 induction, suppression of p53, and increased NF-kB signaling

Although radiotherapy is a mainstay in the treatment of GBM, radioresistance remains a clinical challenge often leading to poor patient prognosis [28]. Since we found that CD146 expression correlated with patient response to therapy (Fig. 1H, I), we next sought to investigate whether CD146 determines radiation sensitivity in our in vitro models. Clonogenic survival assay was performed in GG16-CD146/GFP and control differentiated cells exposed to 2, 4, and 6 Gy γ-rays. GG16-CD146/GFP cells were significantly more resistant to radiation than control cells (Fig. 6A, B). Interestingly, endogenous CD146 (Mw around 120 kDa) expression was also induced in both GG16 and GG16-CD146/GFP cells when exposed to radiation (Fig. 6C). This indicates that CD146 may play a role in regulating the radiation response.

We reasoned that CD146 may regulate radiosensitivity by modulating the DNA damage response (DDR). We found that CD146/GFP overexpressing cells induced a long-lasting activation of both checkpoint kinases CHK1 and CHK2, indicated by prolonged accumulation of phospho(p)-CHK1 and p-CHK2 levels compared to control GG16 cells at 16 h after irradiation with 4 Gy (Fig. 6C). Despite this, apparently similar levels of radiation-induced DNA damage and repair were detected in this isogenic model as shown by a similar γ-H2AX expression pattern. Interestingly, ectopic expression of CD146/GFP in GG16 cells resulted in higher p-MDM2 levels and a striking decrease in p53 expression when compared to control cells. After irradiation, GG16 control cells displayed a time-dependent increase in p53 levels together with a decrease in p-MDM2 at 16 h post-irradiation, while p53 remained low and p-MDM2 high in GG16-CD146/GFP cells. In addition, higher NF-κB levels were detected in untreated GG16-CD146/GFP cells (Fig. 6D). Overall, these results indicate that CD146 can promote radioresistance in GBM cells involving activation of DNA damage responses, suppression of p53, and increased NF-κB survival signaling.

### CD146 activates YAP via the Hippo pathway

**Fig. 7:**
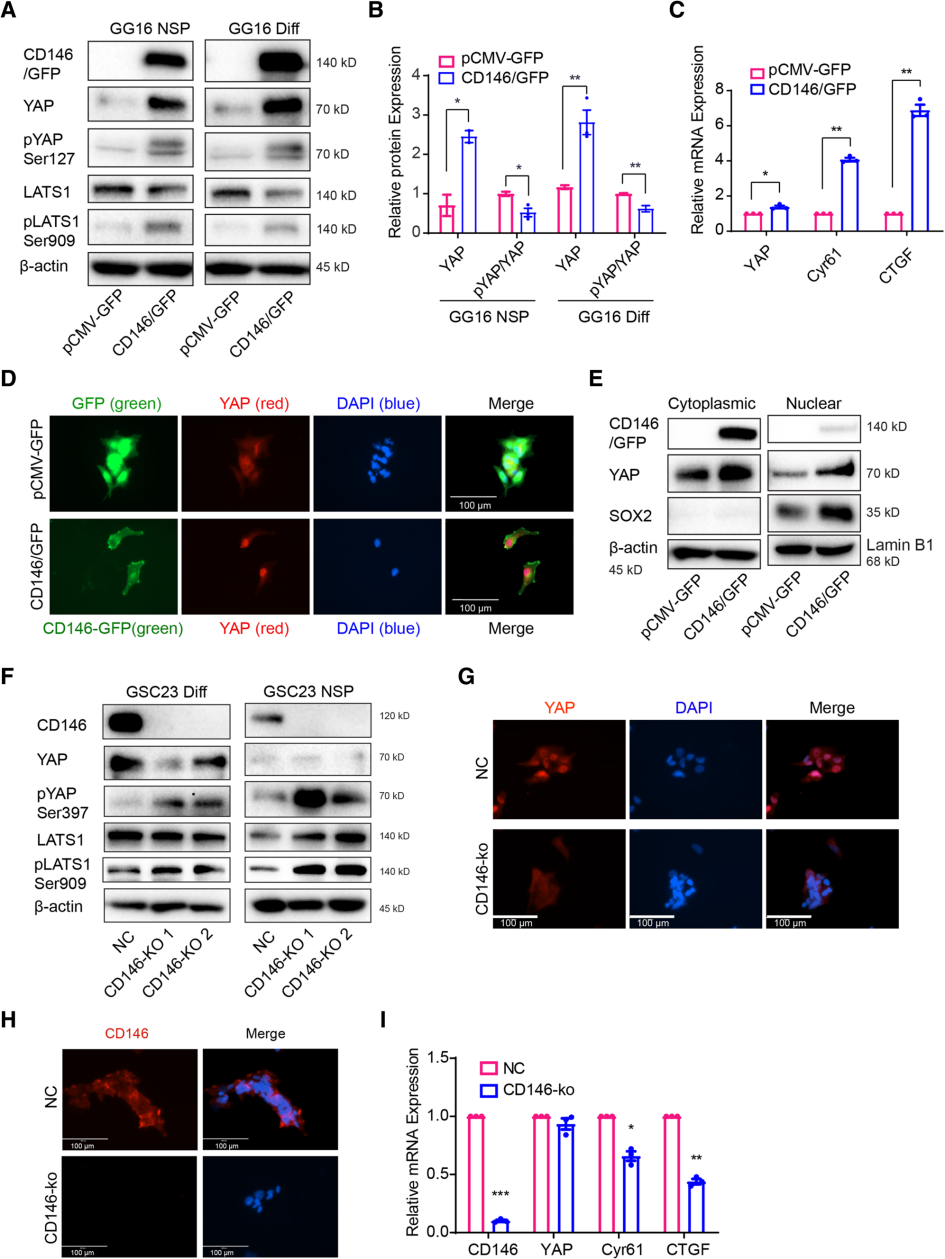
CD146 activating YAP signaling activity. **A** Protein levels of CD146 and Hippo/YAP pathway molecules were determined by Western blotting in GG16 GFP control (pCMV-GFP) and CD146/GFP overexpressing cells under neurospheres (NSP) and differentiated conditions (Diff). CD146 induced YAP expression accompanied by increased phosphorylation of YAP and LATS. **B** Bar graphs showing relative levels of YAP normalized to β-actin (loading control) together with ratio of pYAP (inactive)/YAP (active). The ratio decreased in CD146 overexpressing cells suggesting increased YAP activity. **C** The mRNA levels of *YAP*, *CYR61*, and *CTGF* were detected by qRT-PCR, indicating YAP activation by CD146. **D** Immunofluorescence microscopic images of GG16-GFP or GG16-CD146/GFP cells stained for YAP (red) and nuclei (blue; scale bars = 100 μm); green signal (GFP or CD146-GFP). Nuclear YAP was enhanced by CD146/GFP overexpression. **E** Western blotting of cytoplasmic and nuclear extracts showing levels of CD146, YAP, and SOX2 in serum-differentiated GG16 GFP control and CD146/GFP cells. CD146 increased YAP levels in both fractions. β-actin used as loading control of cytoplasmic fractions and Lamin B1 for nuclear fractions. SOX2 was included as additional control for purity of extracts. **F** Western blots detecting CD146, YAP, and Hippo pathway proteins in serum-cultured and neurospheres GSC23 control and CD146-ko cells. CD146 loss reduces YAP expression and increases pLATS and pYAP levels. **G** Immunofluorescence microscopic images of GSC23 control and GSC23-CD146-ko serum-cultured cells depicting cytoplasmic (inactive) and nuclear (active) YAP (red). Nuclei are counterstained with DAPI (blue). Loss of CD146 reduces nuclear YAP (scale bars, 100 μm). GSC23 control cells showed nuclear YAP, whereas in CD146-ko cells a clear cytoplasmic shift of YAP was detected. **H** Immunofluorescence of differentiated GSC23 or GSC23 CD146 knockout cells stained for CD146 (red) and nuclei (blue; scale bars, 100 μm). Membranous CD146 is detected in control cells, absent in CD146-ko cells. **I** qRT-PCR analyses of YAP target genes CYR61 and CTGF showing reduced expression in GSC23-CD146-ko cells. *p < 0.05, **p < 0.01 by Student’s *t* test

Previously, the expression of CD146 has been reported to be transcriptionally regulated by YAP in hepatocellular carcinoma cells [29]. In order to examine potential regulatory interactions between CD146 and YAP, we first determined their protein levels and found mostly a positive correlation in expression in the GBM models (Supplementary Fig. 3A, B). In addition, in the GBM-cortical assembloid model the expression of YAP was also strongly reduced in CD146-deficient GSC23 cells compared to CD146-proficient controls (Supplementary Fig. 2A and D). Similarly, in GG16-CD146/GFP cells the expression of YAP dramatically increased compared to GG16-GFP control cells (Fig. 7A).

YAP accumulation is known to be negatively regulated by the kinase LATS1, which by phosphorylating YAP induces its cytoplasmic degradation [30]. The expression of LATS1 was reduced by CD146/GFP overexpression and at the same time accompanied by increased pLATS1 and pYAP, whereas total YAP levels remained elevated (Fig. 7A). Quantification of pYAP *vs.* total YAP ratios (Fig. 7B) showed decreased ratios in CD146/GFP overexpressing cells compared to control cells, thus predicting relative higher levels of active nuclear YAP. This was corroborated by an observed dramatic increase in nuclear YAP in GG16-CD146/GFP cells, compared to more dispersed cytoplasmic YAP staining found in GG16 control cells (Fig. 7D).

To further validate subcellular YAP distribution, cytoplasmic and nuclear lysates were prepared from differentiated GG16-GFP control and CD146/GFP overexpressing cells. Western blotting showed increased YAP expression in both cytoplasmic and nuclear fractions in CD146/GFP overexpressing cells, indicating overall higher levels of active YAP compared to control cells (Fig. 7E). Of note, SOX2 was only found in nuclear fractions and levels increased upon ectopic CD146/GFP expression, in agreement with the above described findings.

Nuclear YAP acts as a cofactor of TEAD transcription factors and regulates the expression of target genes, including Cysteine Rich Angiogenic Inducer 61 (*CYR61*) and Connective Tissue Growth Factor (*CTGF*) [31]. Indeed, qRT-PCR analyses showed increased transcript levels of *CYR61* and *CTGF* in GG16-CD146/GFP cells compared to control cells illustrating CD146-dependent YAP activation (Fig. 7C).

The observed regulation of YAP by CD146 was further corroborated in the GSC23-CD146-ko model. In CD146-ko cells the increased levels of pYAP ser397 and pLATS1 ser909 were observed compared to control cells, while total LATS1 expression remained mostly the same (Fig. 7F). These findings suggest that CD146 suppresses the Hippo pathway (reduced pLATS1 and pYAP levels) providing an explanation for increased YAP expression and, conversely, reduced YAP levels in the CD 146-ko cells. Examination of the subcellular localization showed nuclear YAP in GSC23 control cells, whereas in CD146-ko cells YAP was detected in the cytoplasm (Fig. 7G, H). This was associated with reduced transcriptional activation of *CYR61* and *CTGF* in GSC23-CD146-ko cells compared to control (Fig. 7I).

All together these data provide evidence that CD146 can increase YAP nuclear translocation and activation of target genes and identifies the Hippo/YAP signaling pathway as a downstream effector of CD146 in enhancing tumor aggressiveness in GBM.

## Discussion

CD146 is regarded as a tumor-promoting protein in many cancers; however, its activity and function in GBM have been poorly studied. Here, we demonstrated that CD146 is an important regulator of MT, cell invasion, and stemness in GBM and showed a strong stimulatory effect on GSC tumor dissemination in human GBM-cortical assembloids and zebrafish xenografts. In addition, CD146 enhanced radioresistance that was linked with increased survival signaling involving suppression of p53 and an increase in NF-kB activity. Moreover, we identified the transcriptional regulator YAP as a downstream effector of CD146.

Analysis of TCGA/ CGGA databases revealed elevated CD146 expression in GBM compared to normal brain tissue. Moreover, increased CD146 expression correlated with higher glioma grades, IDH-wildtype status and unmethylated MGMT phenotypes. These findings are in agreement with, and an extension of, a previous study showing correlations between CD146 expression and glioma grade [15]. In addition, we found that CD146 expression also correlates with treatment resistance. Thus, CD146 can serve as a biomarker for poor prognosis in GBM.

In GBM neurospheres, we found variable and mostly low expression levels of CD146, which was strongly enhanced upon cell adherence mediated by either ECM or differentiation. Since CD146 is a cell adhesion molecule known to interact with ECM proteins, such as laminins, increased expression of CD146 likely facilitates cell adherence [7, 8, 32]. In addition, CD146 activity has been linked with various processes, including cytokines and growth factor signaling, cell growth, cell–cell communication, inflammatory responses, and EMT [32]. Here, we found that CD146 regulates the mesenchymal status of GBM cells and stimulates cell invasion. TGF-β induced MT and CD146 expression in U-87MG cells and plasmid-based CD146 overexpression in GG16 cells resulted in a mesenchymal phenotype. The opposite was found in generated CD146-deficient GSC23 cells. Our findings in GBM are in line with reports in other tumor types showing that CD146 can induce a mesenchymal phenotype [9, 10].

GBM is characterized by an extensive heterogeneity leading to a poor clinical outcome. Cellular heterogeneity is associated with one of the salient features of GSCs, which endows self-renewal potential, high tumorigenic ability, and resistance to conventional therapy [4]. Recently, Toshio et al*.* reported that CD146 could be regarded as a GSCs marker [33]. Here, we provide functional evidence that CD146 expression enhanced neurosphere formation in GSC models which was accompanied by increased expression of stem cell markers SOX2 and Oct-4. In accordance with our results, the role of CD146 as a marker for normal and cancer stem cells has been shown previously including in mesenchymal stem cells, dental stem cells, and tumor-initiating cells in sarcoma [34–36]. Of interest is also the perivascular niche reported to be involved in GSC maintenance [37] since CD146 is a well-known pericyte marker and may facilitate interactions with GSCs or may stimulate vascular mimicry of GSCs although this remains to be examined [38].

Radiotherapy is part of the standard post-surgery treatment in an effort to eliminate any residual tumor cells. However, intrinsic radioresistance of tumor cells or resistance acquired during radiotherapy leads to recurrent disease and poor survival [3, 39]. We found that CD146 contributes to radioresistance in our GBM models. This is in line with a previous report in which antibody-dependent inhibition of CD146 could enhance IR sensitivity in cervical cancer [40]. It has been reported that GSCs and MT play an important role in radioresistance of GBM cells [28, 41]. Therefore, our finding that CD146 stimulated both a mesenchymal and stem cell phenotype in GBM cells provides a mechanism for radioresistance. Moreover, we have identified downstream molecular mechanisms that can mediate CD146-dependent radioresistance. CD146 overexpressing and control cells demonstrated similar levels of IR-dependent DNA damage and CHK2 activation, whereas clear differences were observed in CHK1 activation, MDM2 phosphorylation, p53 accumulation, and NF-κB activation. It is known that activation of CHK1, CHK2, and NF-κB results in GBM radioresistance and accordingly these pathways likely contribute to CD146-mediated radioresistance [28, 42]. Particularly p53 expression was strongly reduced by CD146 overexpression both in control and irradiated cells, identifying CD146 as a potent suppressor of p53 expression, which may reduce apoptosis activation. The increased levels of phosphorylated MDM2 detected in CD146 overexpressing GG16 cells may enhance proteasomal degradation and provide an explanation for p53 reduction. The more precise way in which CD146 signals to NF-κB and MDM2 remains to be elucidated.

Finally, we identified the transcriptional regulator YAP as a new downstream effector of CD146 signaling. YAP, a transcriptional co-activator, can modulate the expression of various target genes involved in different adaptive responses, such as balancing stem cell proliferation in tissue formation and regeneration, cellular communication via ECM interactions, and mechanotransduction [43]. In cancer, YAP functions as an oncogenic protein involved among others in tumorigenesis, EMT, and metastasis [13, 14, 31, 44]. It is well established that the Hippo pathway can directly regulate YAP whereby activated MST1/2 kinases associate with its scaffolding partners SAV1 and phosphorylate and activate LATS1/2. Subsequently, activated LATS1/2 kinases bind and phosphorylate YAP to prevent nuclear translocation and/or promote cytoplasmic protein degradation [45]. Here, we found that CD146 levels positively correlate with YAP expression. GSC23 cells with relative high levels of endogenous CD146 showed nuclear localized YAP and target gene activation, which was impaired by CD146 knockout. GSC23-CD146-ko cells displayed increased YAP phosphorylation and strong decreased active YAP protein levels. This suggests that CD146 represses Hippo pathway and pYAP leading to increased active YAP levels in the nucleus. Consistently, ectopic expression of CD146 in GG16 dramatically increased YAP protein levels and nuclear transcriptionally active YAP. However, CD146 overexpression in GG16 simultaneously increased pLATS1 and pYAP expressions, which would counteract YAP activation. It could be that the high levels of ectopic CD146 and active YAP trigger a negative feedback that tries to balance YAP activity by activating pLATS1/ pYAP in order to keep cellular homeostasis. The more precise interaction between CD146 and YAP signaling remains to be elucidated and relevance for GBM aggressiveness should be further explored.

In the GBM-cortical assembloid and zebrafish xenograft models we found that CD146 enhances the migratory and invasive properties of GSCs. Interestingly, in zebrafish xenografts CD146 depletion in GSC23 resulted in more condensed and delineated tumor formation and strongly reduced tumor cell dissemination, indicating that the cell adhesion function of CD146 is less important than its cell surface receptor function. Several ligands for CD146 have been identified, including Laminin 411 and 421, Galectin-1 and -3, S100A8/A9, and matriptase, in addition to its found co-receptor function for pro-angiogenic receptors [46]. Thus, the receptor function of CD146 is likely responsible for the observed phenomena, although the ligands that are responsible for CD146-dependent enhancement of tumor aggressiveness remain to be identified.

CD146 located at the cell surface represents an attractive target for therapy. Yang et al. reported that an anti-CD146 monoclonal antibody (YY146) can mitigate GBM aggressiveness in a xenograft mouse model. In addition, 64Cu-labeled YY146 was used for noninvasive positron emission tomography (PET) imaging of orthotopic GBM models [15]. Furthermore, another monoclonal antibody against CD146 (AA98) has been reported to sensitize cervical cancer cells to radiation [40]. Further studies are warranted to explore the possible therapeutic benefit of targeting CD146 in GBM.

In summary, our work identifies CD146 as an important regulator of aggressiveness and radioresistance in GBM and identifies YAP as a potential CD146 downstream effector. Together, this illustrates the potential of CD146 as a target for the development of therapies against GBM.

### Supplementary Information

Below is the link to the electronic supplementary material.

Supplementary file1 (DOCX 3339 KB)

## Data Availability

The authors confirm that all the data supporting the findings of this study are available within the article and supplementary materials. The gene expression and GBM patients’ clinical data are available via the TCGA (The Cancer Genome Atlas, HG-UG133A microarray and GBMLGG RNA-seq data) and CGGA data portal (Chinese Glioma Genome Atlas, http://www.cgga.org.cn/).

## Abbreviations

CD146: Cluster of differentiation 146
DDR: DNA damage response
EMT: Epithelial-to-mesenchymal transition
GBM: Glioblastoma
GSCs: GBM stem cells
IDH: Isocitrate dehydrogenase
iPS: Induced pluripotent stem
IR: Irradiation
MGMT: O6-methylguanine DNA methyltransferase
MT: Mesenchymal transition
MCAM: Melanoma cell adhesion molecule
YAP: Yes-associated protein
