# Supplementary material for: CD146 increases stemness and aggressiveness in glioblastoma and activates YAP signaling
Source: Cell Mol Life Sci. 2022 Jul 5;79(8):398. doi: 10.1007/s00018-022-04420-0 (PMC9256581; doi:10.1007/s00018-022-04420-0)
Supplement: Supplementary file 1 — Supplementary file1 (DOCX 3339 KB) [file 18_2022_4420_MOESM1_ESM.docx]

**Supplementary Information**

**Methods**

**Human cortical organoid generation**

The induced pluripotent stem (iPS) cell line EH1 [19] was cultured using Tesr1 medium (Stem Cell Technologies, Germany, #85850) on matrigel coated plates (Corning, NY, USA, #354277) and passaged every 4-5 days using manual dissociation or ReLeSR (Stemcell Technologies, #5872). iPS cells were differentiated into cortical organoids using the protocol published by the Pasca laboratory [20, 21]. Briefly, iPS colonies that were ready to be passaged were incubated with dispase (0.35 mg/ml, Thermo Fisher Scientific, #17105041) until detachment. Detached colonies were then cultured in hPSC medium on to ultra-low- attachment plates (Sigma-Aldrich, #CLS3814-24EA) for the first 5 days. hPSC medium consisted of DMEM/F12 (Thermo Fisher Scientific, 11330-032), 20% KnockOut serum replacement (Thermo Fisher Scientific, #10828010), 1% non-essential amino acids (Thermo Fisher Scientific, #11140035), 0,5% Glutamax (Thermo Fisher Scientific, #35050038), 2-mercaptoethanol (0.1mM, Sigma-Aldrich, #M3148), 1% penicillin/streptomycin (Thermo Fisher Scientific, #15140-163), 0.1% Mycozap Plus-PR (Lonza, Breda, Netherlands, #VZA-2021), dorsomorphin (5µM in DMSO; Sigma-Aldrich, P5499-5MG), and SB-431542 (10 µM in ethanol, Tocris, Bristol, UK, #1614) and was supplemented with ROCK inhibitor Y-27632 (10 µM, Bioconnect, Huissen, Netherlands, #D455443) for the first day of organoid formation. From day 6 onwards, cortical organoids were cultured in neural differentiation medium containing Neurobasal-A medium (Thermo Fisher Scientific, 10888022), 2% B27 without vitamin A (Thermo Fisher Scientific, #12587010), 1% Glutamax (Thermo Fisher Scientific, #35050038), 1% penicillin/streptomycin (Thermo Fisher Scientific, #15140-163), 0.1% Mycozap Plus-PR (Lonza, #VZA-2021) supplemented with with fibroblast growth factor-2 (20ng/ml, Preprotech, London, UK, #100-18B) and epidermal growth factor (20ng/ml, Sigma-Aldrich, #E9644) until day 25 and brain derived neurotrophin factor (20ng/ml, PeproTech, 450-02) and neurotrophin-3 (20ng/ml, PeproTech, #450-03) from day 25 onwards.

**GBM-cortical assembloid model**

Around 35 day-old cortical organoids were fused with GFP-expressing GSC23 control and CD146-ko neurospheres (diameter of approximately 400 μM, n=3) to form the here called GBM-cortical assembloids in an Eppendorf tube for 15 hours in 50% neurosphere culture medium and 50% neural differentiation medium. GBM-cortical assembloids were further maintained in ultra-low-attachment plates (Corning, #CLS3473-24EA) up to 96 hours. Confocal fluorescent microscopy was performed by using the Zeiss LSM 7 MP microscope. Invasion was determined by imaging GBM-cortical assembloids at 15 and 96 hours after fusion using the EVOS XL Core Cell Imaging System (Thermo Fisher Scientific) and determining the area of the cortical organoid that had become GFP+ between 15 and 96 hours after fusion. For further analyses, GBM-cortical assembloids were fixed in 4% paraformaldehyde (Electron Microscopy Sciences, Wageningen, Netherlands #15713) for 1.5 hours, washed with PBS and incubated in 30% w/v sucrose (Merck Life Sciences, Darmstadt, Germany, #573113) for 48 hours. GBM-cortical assembloids were then embedded in KP-Cryo Compound (Klinipath, Duiven, Netherlands, #1620C), frozen and sectioned in 10 µm slices for immunofluorescent staining with CD146/MCAM, MAP, SOX2 and YAP antibodies (see Supplementary Table S1) followed by microscopy.

**Zebrafish xenograft experiments**

GSC23-WT or GSC23-CD146KO accutase dissociated cells were stained with CellTracker™ Green CMFDA Dye (Invitrogen, Netherlands) for 30 min in the incubator and washed twice with cold PBS. Subsequently, cells were gently resuspended in Neurobasal A-medium with 2% (w/v) polyvinylpyrrolidone (PVP) at 0.125 x 10^6^/ul concentration and were kept on ice until transplantation. Embryos at 1 dpf were manually dechorionated, anesthesized with tricaine and transferred to an agarose dish with single slots. Cell suspension was back-loaded into pulled thin-wall capillaries with filament (TW100F-4; World Precision Instruments) and the capillary was inserted through the yolk to reach the PVS (1). Here, 1-4 injection pulses were applied using a pneumatic picopump (PV820, WPI) such that a visible bolus of GSC23 cells was present in the PVS space. After completion of the xenografts, the embryos were transferred to a clean dish with E3 medium and cultured at 34°C. Embryos were examined within 4 hours post-transplantation and sorted into groups containing embryos with cells only in PVS or embryos also containing cells in circulation. Embryos with visibly few cells present or cells only present inside the yolk sac were discarded. For some experiments, individual embryos were repetitively photographed and kept in individual wells of a 24 well-plate until fixation. Images of live embryos were taken with a Leica MZ FLIII fluorescence stereomicroscope equipped with a Leica DFC3000G digital camera. The data presented are results from 3 independent experiments.

**Immunofluorescence analyses of zebrafish xenografts**

Zebrafish xenografts were fixed in 4% paraformaldehyde (Fisher Scientific) overnight at 4°C and stored in PBS at 4 °C for maximum 2 weeks before immunostaining. Embryos were permeabilized for 45 minutes using 10 μg/ml Proteinase K (Sigma-Aldrich, #3115801001) in PBS, post-fixed for 20 minutes with 4% PFA and blocked with 3% BSA (Sigma-Aldrich, #A9647) in PBS with 0.3% Triton-X. Embryos were incubated with primary antibody solution (1:100 dilution) for at least 16 hours at 4°C. After washing and blocking, embryos were incubated overnight at 4°C with secondary antibody (goat anti-mouse Alexa-488 (Life technologies #A11029, 1:500)). After washing, nuclei were counterstained with DAPI in PBS for at least 1 week at 4°C. Subsequently, embryos were mounted in a coverslip-bottom petridish (Ibidi) in low-melting point agarose and imaged with a 20x (HC PL APO CS N.A. 0,50 air objective on a Leica SP8 confocal microscope or with a 40x (HC PL APO CS2 N.A. 1,3) oil objective on a Leica SP8X DLS microscope in confocal mode at a z-interval of 1.2 to 2 μm and a xy pixel size of 379 to 437 nm. Maximum z-projections were generated and adjusted for contrast/brightness with Fiji (2). For the categorization of tumor morphology, the green channel images were converted to inverted grey LUT, randomly coded and scored blindly by two investigators.

| **Table S1. Proteins and description of corresponding antibodies** | | | |
| --- | --- | --- | --- |
| **Antibodies** | **Vendor** | **NO.** |  |
| CD146 | Cell Signaling | 13475 |  |
| Nucleoli | Abcam | Ab190710 |  |
| YAP | Cell Signaling | 14074 |  |
| p-YAP Ser127 | Cell Signaling | 13008 |  |
| p-YAP Ser397 | Cell Signaling | 13619 |  |
| LATS1 | Cell Signaling | 3477 |  |
| pLATS1 Ser909 | Cell Signaling | 9157 |  |
| β-Catenin | BD transductor | 610154 |  |
| ZEB1 | Novusbio | NBP1-05987 |  |
| Twist | Abcam | Ab50887 |  |
| Fibronectin | BD transductor | 610077 |  |
| N-cadherin | Cell Signaling | 13116 |  |
| SOX2 | R&D System | MAB-2018 |  |
| Oct-4 | Cell Signaling | 2750 |  |
| pCHK1 | Cell Signaling | 12302S |  |
| pCHK2 | Novusbio | NB100-92502 |  |
| γ-H2A-X | Cell Signaling | 9718 |  |
| MDM2 | Santa Cruz | Sc-965 |  |
| pMDM2 | Cell Signaling | 3521 |  |
| P53 | Cell Signaling | 2324 |  |
| MAP2 | Cell Signaling | 8707 |  |
| pNFκB | Cell Signaling | 3033 |  |
| CD146 | Abcam | Ab75769 |  |

**Table S2. Primers for qRT-PCR**

|  | Sequences (5' to 3') |
| --- | --- |
| CTGF | F CCAATGACAACGCCTCCTG |
|  | R TGGTGCAGCCAGAAAGCTC |
| Cyr61 | F AGCCTCGCATCCTATACAACC |
|  | R TTCTTTCACAAGGCGGCACTC |
| YAP | F GATCCCTGATGATGTACCACTGCC |
|  | R GCCATGTTGTTGTCTGATCGTTGTG |
| CD146 | F AGCTCCGCGTCTACAAAGC |
|  | R CTACACAGGTAGCGACCTCC |
| β-actin | F GAGACCTTCAACACCCCAGCC |
|  | R AATGTCACGCACGATTTCCC |

**Supplementary Figures**


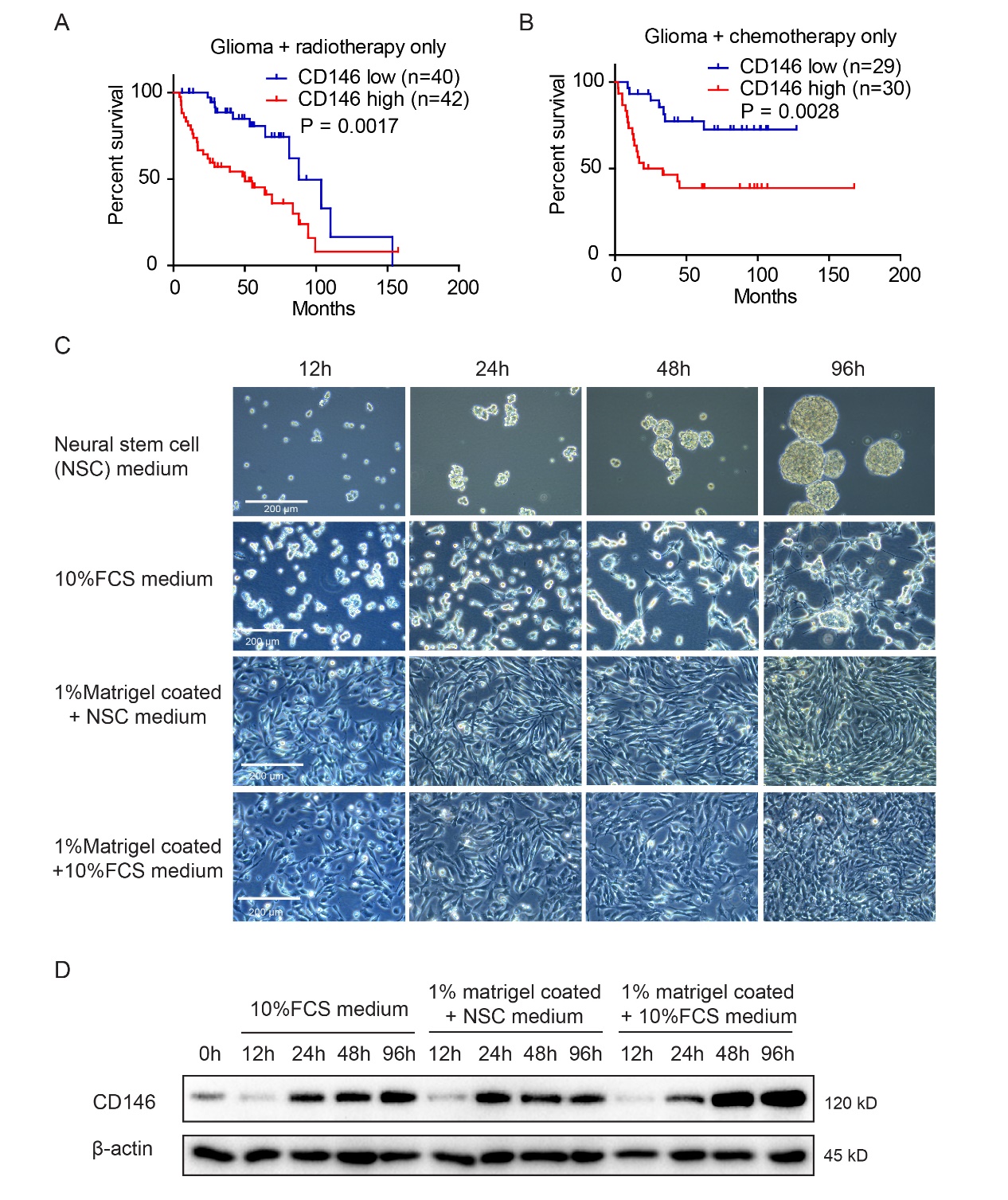


**Supplementary Fig. 1**

**CD146 expression in GBM neurospheres is enhanced upon cell adherence.**

**A-B.** Kaplan–Meier plot showing overall survival between high CD146 and low CD146 expression of Glioma patients who only received radiotherapy (A) or only received chemotherapy (B). **C.** Representative phase contrast microscopy images (scale bars = 200 μm) of GSC23 cell morphology during time (12h, 24h, 48h, 96h) after seeding in serum-free neural stem cell (NSC) medium, 10%FCS medium, 1% Matrigel-coated wells in serum-free NSC medium and 1% Matrigel-coated wells in 10%FCS medium. **D.** Western blots showing cell adherence- and differentiation-dependent enhancement of CD146 protein expression during time.


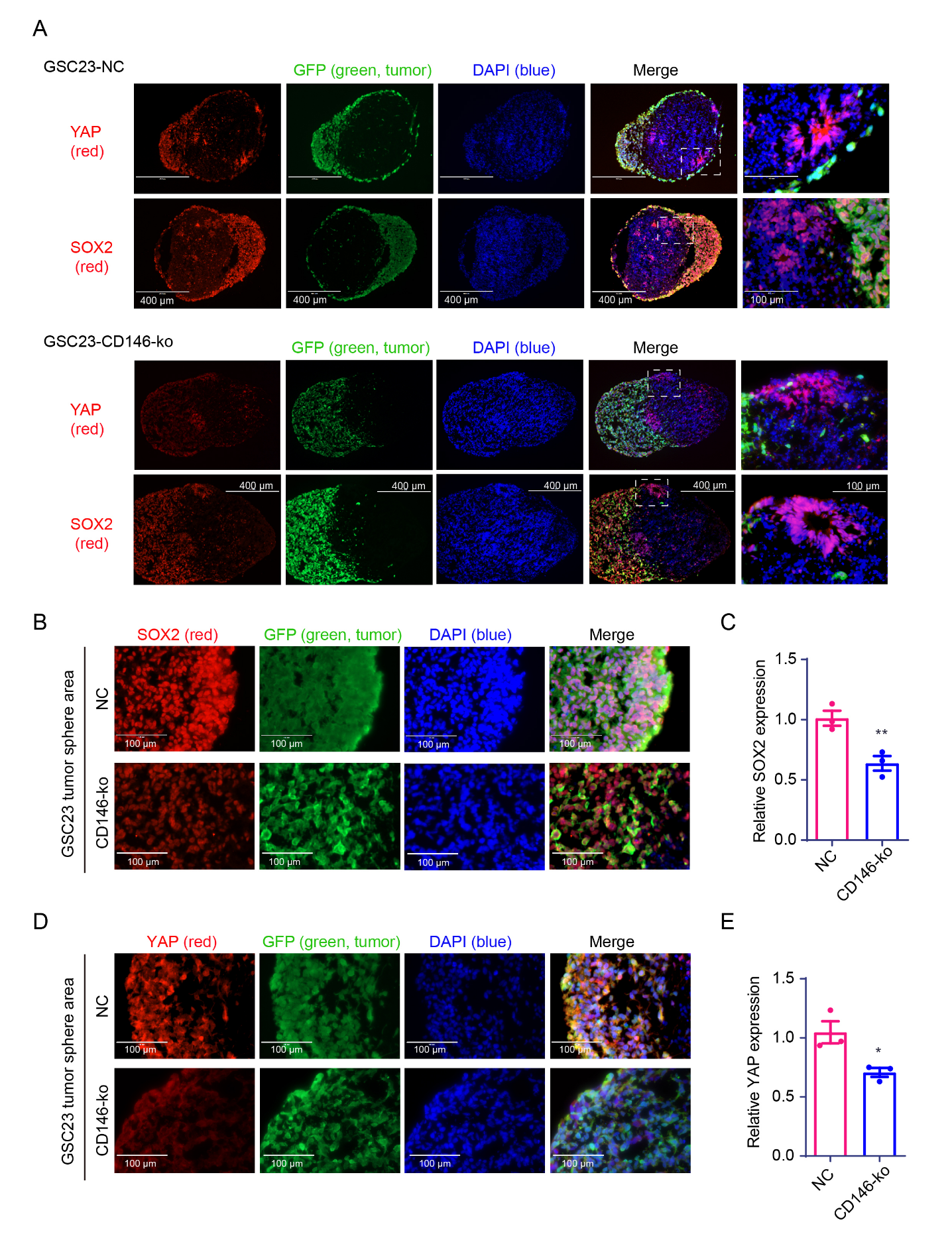


**Supplementary Fig. 2**

**CD146 promotes GSC23 migration/ invasion in GBM – cortical assembloid model.**

**A.** Immunofluorescence microscopic images of GFP-labelled (green: GSC23 cells) GSC23 and GSC23-CD146-ko GBM– cortical assembloids) stained for YAP and SOX2 (labeled red). Nuclei (blue) were stained with DAPI. scale bars = 400μm (left), scale bars = 100 μm (right). **B.** Immunofluorescence microscopic images showing enlargements of GFP-labelled GSC23 and GSC23-CD146-ko area stained for SOX2 (labeled red) to appreciate differences in expression. Nuclei (blue) were stained with DAPI. scale bars = 100 μm. **C.** Quantification of SOX2 relative expression in GFP positive GSC23-NC and GSC23-CD146-ko tumor sphere area. **D.** Immunofluorescence microscopic images showing enlargements of GFP-labelled GSC23 and GSC23-CD146-ko area stained for YAP (labeled red) to appreciate differences in expression. Nuclei (blue) were stained with DAPI. scale bars = 100 μm. **E.** Quantification of YAP relative expression in GFP positive GSC23-NC and GSC23-CD146-ko tumor sphere area. *p<0.05, **p<0.01 by Student’s t test.


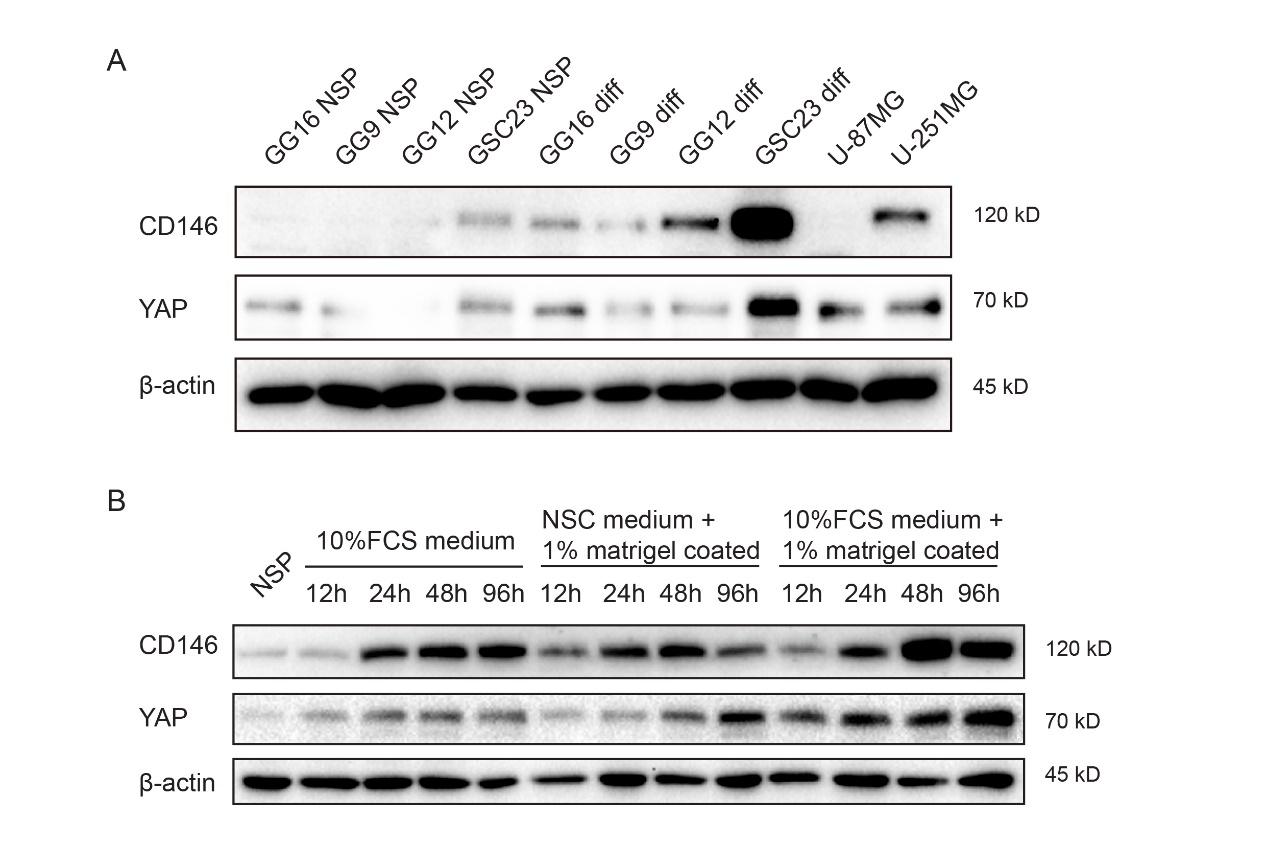


**Supplementary Fig. 3**

**Positive correlation between CD146 and YAP expression.**

**A.** Western blots showing expression of CD146 and YAP in the indicated GBM cells cultured in serum. **B.** Western blots showing CD146 and YAP protein levels in GSC23 cells when cultured in serum-free neural stem cell (NSC) medium, 10%FCS medium, 1% Matrigel-coated wells in serum-free NSC medium and 1% Matrigel-coated wells in 10%FCS medium.
